# Validation of a Tablet as a Tangent Perimeter

**DOI:** 10.1167/tvst.5.4.3

**Published:** 2016-07-14

**Authors:** Algis J. Vingrys, Jessica K. Healey, Sheryl Liew, Veera Saharinen, Michael Tran, William Wu, George Y. X. Kong

**Affiliations:** 1 Department of Optometry & Vision Sciences Melbourne School of Health Sciences, University of Melbourne, 3010, Victoria, Australia; 2 Centre of Eye Research Australia, 32 Gisborne St East Melbourne, Victoria, Australia

**Keywords:** iPad, automated perimetry, visual field, simulated scotoma

## Abstract

**Purpose:**

To describe a tangent perimeter developed on an Apple iPad (Melbourne Rapid Field, MRF).

**Methods:**

The MRF assays 66 locations over 28° × 18° by having the patient vary fixation. Spot size and background luminance are paired to yield constant thresholds across the field. Spot locations were selected after analysis of 360 patient records. The capacity of the MRF to detect defects was verified in five participants (age 22–28 years) by simulating four common losses: central, arcuate, quadrant, and hemianopia. We also consider the effect of: myosis, blur (+3 DS), viewing distance (25–75 cm), ambient light (4–600 lux), and retest repeatability (1-week apart) on thresholds. Group means [SEM] are compared by Student's *t*-test and repeatability returned from Bland-Altman analysis.

**Results:**

We found a 5 cd.m^−2^ background replicates the Weber fraction produced by a Humphrey spot shown at 35 dB. Our variable size gives constant thresholds (29.6 [0.2] dB) across all locations. Altering viewing distance (25 cm = 29.8 [0.9] dB; 75 cm = 28.9 [0.6] dB) and ambient lighting (4 lux, 29.8 [0.8] dB; 600 lux, 29.5 [1.0] dB) did not affect threshold although screen reflections must be avoided. Myosis (−1.2 dB) and blur (−1.5 dB) will reduce sensitivity (*P* < 0.05). Simulated defects with a mean defect (MD) of −3.3 dB are detected by the MRF. The Coefficient of repeatability was 9.6% (SD ∼2.9 dB) in normal regions and 48.1% (SD ∼8.0 dB) in areas of simulated scotoma.

**Conclusions:**

Tablet technology can return efficient and reliable thresholds to 30° as a tangent perimeter.

**Translational Relevance:**

The MRF will allow testing at a bedside, at home, in rural or remote areas, or where equipment cannot be financed.

## Introduction

Tablets such as the iPad (Apple, Inc., Cupertino, CA) have a large dynamic luminance range, 8-bit luminance control, and high spatial resolution that makes them suited as low-cost, portable, tangent-perimeters.^1,2^ Their portability allows vision testing in unusual situations, such as at a bedside, at home, in a clinic waiting room, or in rural and remote areas. Moreover, the ubiquity of tablet devices raises the possibility that patients could perform unsupervised perimetric tests as part of a home-monitoring program. Home-monitoring has been trialed successfully in patients having age-related macula degeneration (AMD) and found to provide better outcomes than standard clinical reviews.^3^

Tablet technology, however, has a lower luminous output than do commercial perimeters so it is not clear that it can provide similar levels of stimulation as do conventional devices. The calculations of Tahir et al.^1^ derived for a background luminance of 214 cd.m^−2^ (half maximal output) indicate that an iPad3 should have adequate capacity to measure increment contrast thresholds over a 1.8 log unit range similar to a Pelli-Robson chart. This suggests a reasonable potential but the prospect that an iPad3 can test foveal thresholds, typically approximately 35 dB, is not apparent from this calculation. The capacity of tablet technology was formally tested by Wu et al.^4^ who report that their iPad3 gave reliable and precise foveal thresholds at low background luminances. However, it is not clear whether tablet devices can be modified to return robust and efficient visual field thresholds at higher background luminances or at peripheral locations given the large changes in luminance that have been measured away from the center of a tablet screen.^1^ It is worth noting that even in the presence of these large luminance changes the authors concluded that contrast remains relatively stable.^1^

We developed the Melbourne Rapid Fields (MRF) as a tangent perimeter on an iPad platform (Apple, Inc.) with the aim of sampling central and peripheral visual fields using fast thresholding methods. This has been part of a continuing development that has seen the early release of a screening application (app) called Visual Fields Easy (VFE), which has shown promising capacity in the field compared with diagnosis based on a Humphrey 24-2 SITA-Standard outcome (HFA).^5^ In that screening application the VFE used a Goldman size V target and a maximum luminance stimulus shown on a 10 cd/m^−2^ (30 apostilb) background. Coupling this finding with the preceding paragraphs provides indirect evidence that tablet technology can be applied to test thresholds across the visual field.

The purpose of the present manuscript is to give an overview of the latest implementation, MRF, which performs fast thresholding on an iPad at various test locations within 30° of fixation. It will also consider the capacity of this tangent perimeter to detect simulated scotoma and explore those factors that can impact on perimetric outcomes to determine how robust this test is to variations in ambient conditions. Consistent with the foveal iPad test reported by Wu et al.^4^ we find the MRF provides reliable and accurate outcomes that can detect shallow simulated scotoma.

## Methods

### Developing a Tablet as a Tangent Perimeter

Despite the tablet having a modest luminance range and 8-bit control, we find it performs remarkably well in assaying human thresholds. This finding should not be surprising as it is consistent with the large literature that reports human thresholding can be achieved using cathode ray tube technology (e.g., Grisby et al.^6^) where low luminances (<120 cd.m^−2^) and limited luminance resolution can exist.^7^ In their tablet implementation, Wu et al.^4^ used an iPad3 for foveal thresholding at a background luminance of 1.27 cd.m^−2^ and maximum spot luminance of 318 cd.m^−2^ in order to simulate a commercial microperimeter. They found an average foveal threshold of 25.7 (2.4) dB (mean [SD]) in 30 elderly participants (mean [SD] age 70.9 [8.2] years). They report that the tablet returned similar outcomes to those found with a retina-stabilized microperimeter (26.1 [2.4] dB) producing a surprising outcome for a free-space device.

One of the challenges in developing a tablet perimeter is that the decibel scale used to describe outcomes is derived from the light output of the test equipment and does not reflect human physiology. Schiefer et al.^8^ define perimetric decibel as 10*log(Lr/Li) where Lr is the maximum luminance and Li is the smallest incremental step from the background. Using this definition, Wu et al.^4^ report an operating range of 31 dB for an iPad3 whose maximum output was 318 cd.m^−2^. For the iPad3 used by Tahir et al.^1^ (background luminance = 214 cd.m^−2^, max luminance = 428 cd.m^−2^) their Figure 2 shows a range of increment contrast modulation of approximately 1.9 log units or 19 dB. Others report that an iPad operating at midrange (150 cd.m^−2^) can return 23 dB output with bit-stealing.^9^ These comparisons are instructive as, even in the presence of the same equipment, the decibel scales differ substantially due to different background levels, which regulate the step size available to the user by way of a gamma function. A commonly used perimeter, the HFA, has a maximum output of 3183 cd.m^−2^ and gives an operating range of 50 dB with a background of 10 cd.m^−2^ and step of 0.032 cd.m^−2^. Although the HFA appears superior to the iPad from its larger decibel capacity, both devices can measure human thresholds. What is needed for a sensible comparison is to consider these devices in terms of human performance as well as the equipment related decibels to define output, and in the following we will do so by developing the relationship between equipment-specific decibels and the human increment contrast threshold or Weber fraction.

**Figure 1:**
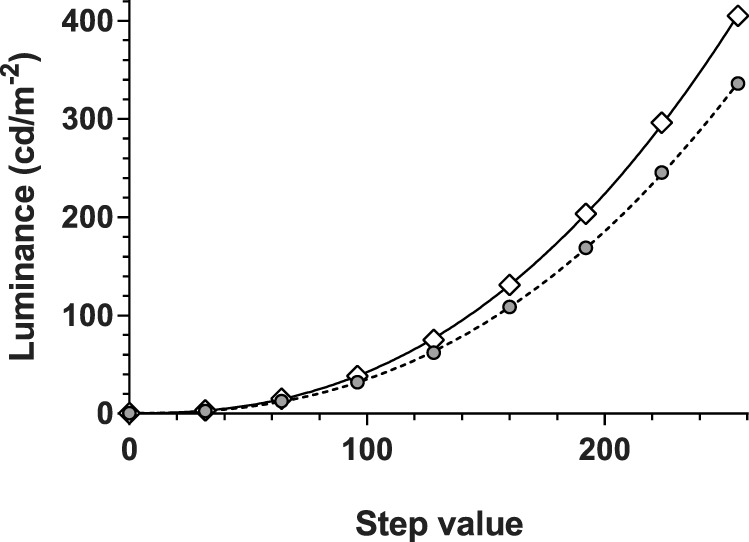
Luminance calibration of two iPad tablet screens: iPad2 (*gray circles*) and iPad3 (*unfilled diamonds*). Both return similar gamma functions (2.4, *lines*) but the iPad2 tablet is consistently dimmer than is the iPad3 due to its lower amplification. This results in a dimmer background level for testing on the iPad2 (4.2 vs. 5.0 cd.m^−2^) but has no practical impact on the dynamic range (30.8 vs. 31.4 dB) as the smaller steps are proportionately scaled by the higher amplification constant.

**Figure 2:**
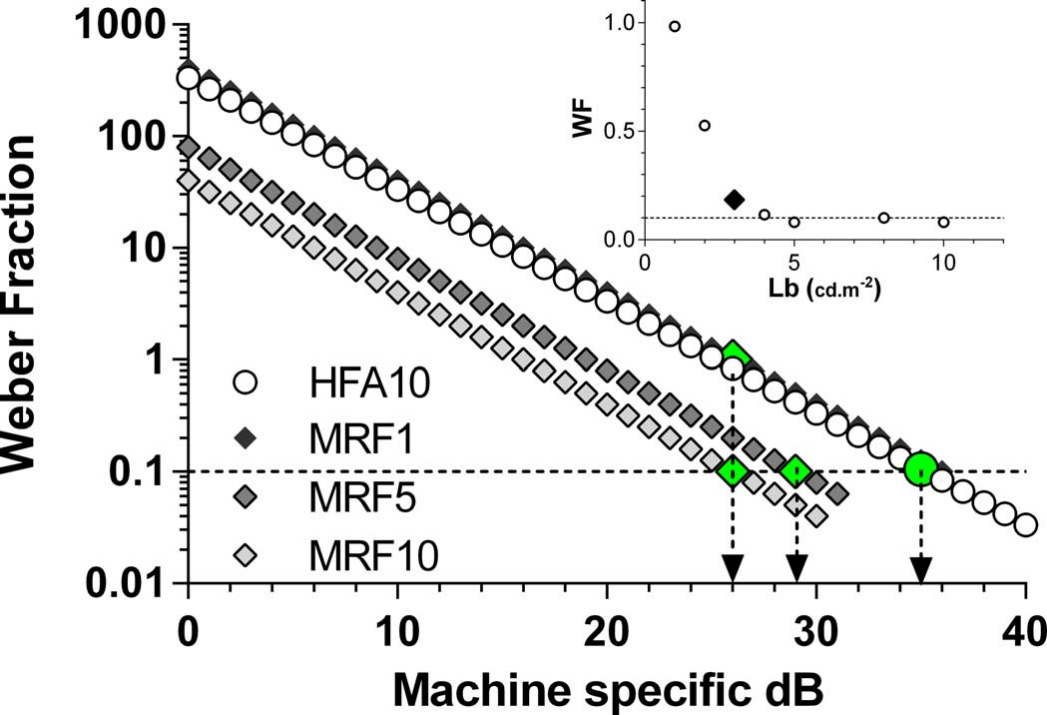
The relationship between equipment specific decibel and the Weber Fraction, on a semi-log plot. Each curve (*line* on this scale) has been calculated at 1-dB steps (*symbols*) as detailed in the text. The *circles* show the relationship for the HFA with the *large filled circle* (and *down arrow*) locating a threshold of 35 dB: note this gives a Weber Fraction of 0.1 as indicated by the *dashed horizontal*. The relationships for the tablet (MRF) at several background luminances (1, 5, 10 cd.m^−2^) are represented by *diamonds* with average normal thresholds indicated as *large* (*filled*) *symbols* and *down arrows*. The 5 and 10 cd.m^−2^ backgrounds yield a Weber fraction that is similar to 35 dB of the HFA (0.1). The 1 cd.m^−2^ background results in reduced sensitivity. The inset plots average Weber fraction (*y* axis) returned from MRF thresholds measured in pilot trials as a function of adapting luminance (*x* axis). Backgrounds less than 3 cd.m^−2^ (*large filled diamond*) produce elevated Weber fractions (reduced retinal sensitivity).

### Expressing the Output of Tablet Technology in Units Relevant to Human Vision

In order to calculate the Weber fraction, one needs to know the maximum luminous output of a tablet and the minimal spot increment that can be produced from that usual background. This information can be achieved by measuring the gamma function. Tahir et al.^1^ have determined the luminous output for three different tablets including an iPad3. They find that their iPad3 gave a maximal output of 428 cd.m^−2^ with a gamma of 1.9. The gamma derived by Tahir et al. (their Equation 3^1^) describes luminous output as a function of voltage input, but voltage is not readily determined from an iPad, although it is related to the 8-bit steps.

For this reason, we determined the gamma function of two tablets (iPad2 and iPad3) and expressed gamma (g) with a formula that describes luminous output (L) in terms of an amplification factor (A), which scales voltage to the video board (8-bit) steps (X), and a ‘dark light' (DL) term (see Equation 13 of Metha et al.^10^) here L = DL + A*X^^g^. Calibration was performed over eight equally spaced steps (average of 4 readings) over the 8-bit range, using an IL1700 radiometer with a photopic filter (head: SED033; International Light Technologies Inc., Peabody, MA).

Both iPad versions returned common gamma and dark light parameters (Fig. 1: g = 2.4; DL = 0.45) but the amplification parameter (A) was significantly larger in the iPad3 (0.00063 vs. 0.00052, *P* < 0.05). This means that each step of the 8-bit controller results in a greater luminous output in the iPad3 device consistent with the measured ranges of 0.46 to 414 cd.m^−2^ compared with the iPad2 device, 0.67 to 346 cd.m^−2^ (Fig. 1). Given the common gamma and lower amplification (A), the iPad2 will yield smaller luminance steps over its 256 levels, thereby producing better luminance resolution along its 8-bit range despite the lower maximum luminance. This difference in step size produces a similar decibel range in both tablets (iPad2 = 30.8, iPad3 = 31.4 dB) demonstrating the difficulty in interpreting machine specific decibel scales in terms of human performance. It is apparent that both versions of the tablet are capable of producing approximately 31 dB of stimulus control consistent with the claim of Wu et al.^4^

Next, we consider how to express iPad performance in terms of a contrast metric (Weber fraction or Li/Lb, where Li is the threshold increment and Lb is the background luminance) that can be applied to human performance. Figure 2 plots the relationship between machine-specific decibel and the Weber fraction at 1-dB steps; the increment contrast (Weber fraction) was determined from, the attenuation (dB) of maximum luminance, divided by the background luminance. Typical thresholds measured for the iPad3 at different background luminances are also shown from pilot trials as large symbols in Figure 2. Note that the maximum luminances of the tablets produce outcomes that terminate earlier (∼31 dB) than does the HFA in Figure 2 (HFA is shown truncated at 40 dB). Also note that any increase in background luminance results in a leftward shift of the curve (curves appear as lines on the semi-log plot of Fig. 2) leaving a reduced capacity (lower dB range) for testing abnormal locations, which is an undesirable attribute for any perimeter. Thresholds obtained by testing on different backgrounds over a range of 1 to 10 cd.m^−2^, generate the data of Figure 2 inset, where we find that backgrounds less than 3 cd.m^−2^ give an increased Weber fraction.

### Choosing a Background Luminance

The Imaging and Perimetry Society^11^ suggest that adapting backgrounds should lie in the range of 1 to 10 cd.m^−2^ and we find that backgrounds less than 3 cd.m^−2^ have elevated Weber fractions. For this reason we chose 5 cd.m^−2^ as the background luminance for the MRF as this gives a good operating range (∼31 dB) at the highest retinal sensitivity (Weber fraction) similar to the 35-dB value of the HFA (Fig. 2). The gamma calibration indicates that the setting that yields a background of 5 cd.m^−2^ for iPad3 returns 4.2 cd.m^−2^ in the iPad2. Figure 2 (inset) shows that this will give a Weber fraction similar to 35 dB of the HFA (0.1). The inset plots average Weber fraction (*y* axis) returned from MRF thresholds measured in pilot trials as a function of adapting luminance (*x* axis). Backgrounds less than 3 cd.m^−2^ (large black diamond) produce elevated Weber fractions (reduced retinal sensitivity). The small difference in background between iPaD2 and iPaD3 will not influence outcomes as thresholds are constant at backgrounds greater than 3 cd.m^−2^.

Given the app will be used as a free space test, the screen of the tablet will be subject to the effects of external light as can occur if testing were to take place in a room with ceiling lights on. So apart from providing an adapting source, the background acts to shield and dilute the external light that is reflected off its surface. This veiling glare can interfere with spot detection. Underlying our choice of background luminance, were measurements of the amount of veiling glare produced by standard office ceiling luminaires (600 lux) measured at the screen of the tablet. We find that this ranged from 1.4 to 3.2 cd.m^−2^ when measured from the location of the subject's eye. This will add to the 5-cd.m^−2^ background of the tablet, producing an effective background of 6.4 to 8.2 cd.m^−2^. Figure 2 shows that changing the effective background with this veiling glare will shift performance to the left and reduce the dynamic range of the test, this effect is small for the levels concerned and will modify threshold by 1 to 2 dB at most. It should be noted that although these calculations indicate that the perimeter can work in the presence of room lights, we recommend that it be administered in a dimly lit room to retain its maximum dynamic range and, to avoid the high veiling glare produced by direct reflections from a bright light source, window or door (see later). These effects will be particularly noticeable at different screen locations.

### Thresholding Logic

**Figure 3:**
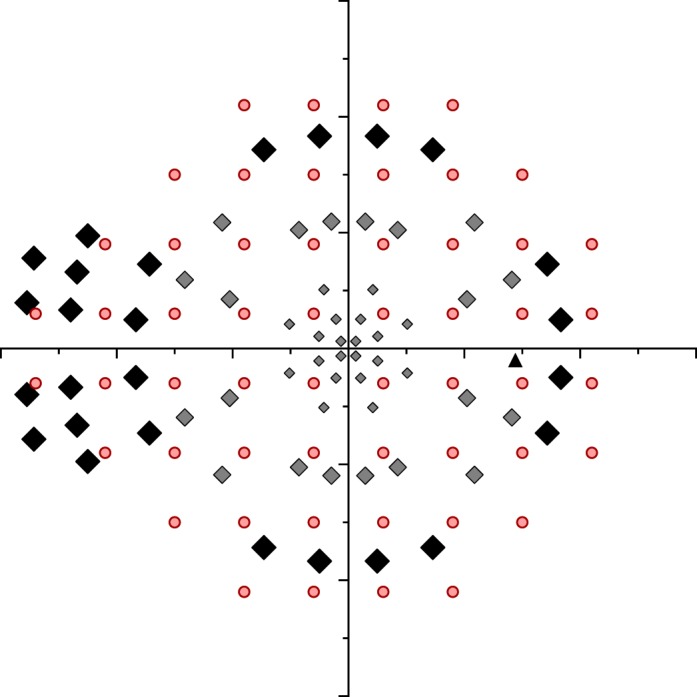
Locations used for testing the Right eye by the MRF (*small gray diamonds*, macula: *medium gray diamonds*, paramacula: *large black diamonds*, peripheral 30°) compared against the HFA 24-2 pattern (*circles*). The blind spot location is shown as a *black triangle*. The variable size of the MRF test spot has been shown schematically by increasing the symbol size and is not accurately scaled.

The steps used by the MRF were scaled to range from 0 to 30 dB over seven discrete levels. Adopting seven levels retains estimates within the capacity of the 8-bit output and can be justified in terms of sampling efficiency. Optimizing sampling efficiency recognizes that threshold variability increases with reduced sensitivity, so step size needs to increase. The conclusion is that seven to eight steps are optimal to sample over a range of 30 dB.^12^ We have adopted a three-presentation binary Baysean protocol to yield eight steps (2^3^) across the 30-dB range (Zippy Estimation by Sequential Testing, ZEST). Our thresholding approach is a binary logic that commences at a level (probability density function [PDF]; see Fig. 1 of Vingrys and Pianta^13^) that is easily visible to 97.5% of normal observers (17 dB). This starting value has the advantage that it provides reinforcement of the task in regions of normal sensitivity, because it is easily seen, and can be used as a screening level for regions with abnormal sensitivity. The rest of the decision tree sequence is predetermined by a modified ZEST procedure^14^ (see Fig. 3 of Vingrys and Pianta^12^). For the MRF implementation, we have assumed that our observers were ‘reliable', knowing full well that they are not. We believe that doing so is justified by the small number of false responses returned by the majority of patients, which in practice is less than 5% (see later where we find no change in 97% of retested values) and that, this approach requires fewer presentations than does one that continues testing after polling a false response early in a test sequence in an otherwise reliable person (see Fig. 2 of Phipps et al.^15^). We deal with cases of lapses in concentration, by retesting points found to be removed from their neighbors. In doing so, we acknowledge that some people will be unreliable during testing but we believe that such people will frustrate endpoint estimates from any test logic. However, one benefit of our approach is that it is rapid requiring three steps for an endpoint, so retesting unreliable locations or unreliable patients becomes a preferred option to continuing with the thresholding processes for a long time in an effort to rectify false response.^15^ The modified ZEST used in our implementation varies the slope of the likelihood function with sensitivity rather than using a fixed slope.^14^ The population PDF and the likelihood functions associated with a response matrix were derived by reanalysis of 17,390 threshold determinations reported by Vingrys and Pianta^13^ scaled to 30 dB. The ZEST logic was then applied to yield a binary decision tree, which gave eight potential end-points (2^3^) that end up being spaced by approximately 4 to 6 dB across the available range of 0 to 30 dB. The MRF implementation has adopted seven of these eight outcomes by collapsing the lower three into two levels, as these were found to be closely spaced. These levels are: 0, 6, 12, 17, 22, 26, and 30 dB.

### The Test Grid, Spot Size, and Testing Peripheral Locations

The MRF test pattern has either a modified 24-2 grid or a radial orientation centered at fixation. In the radial pattern, 66 locations are used, sited on the 8°, 15°, 22°, 45°, 68°, and 82° meridians from the nasal horizontal. Figure 3 shows the grid that includes 20 spots in the central region at 1° (0.9°), 3° (2.7°), and 6° (5.5°) to identify central defects (values in parentheses specify the exact location). A further 20 points are located in the paramacula region at 10° (10.5°) and 14° (14.2°) eccentricity to test paramacula, arcuate, and neurologic regions and a final 26 peripheral points are located at 18° (18.4°), 24° (23.9°), and 28° (27.6°) to test the nasal step and peripheral retina. A subset of these locations are similar to those reported by Wang and Henson^16^ as optimal for glaucoma testing and our simulation trials (Fig. 9) indicate that this grid does a good job in picking up a variety of early simulated defects.

**Figure 4:**
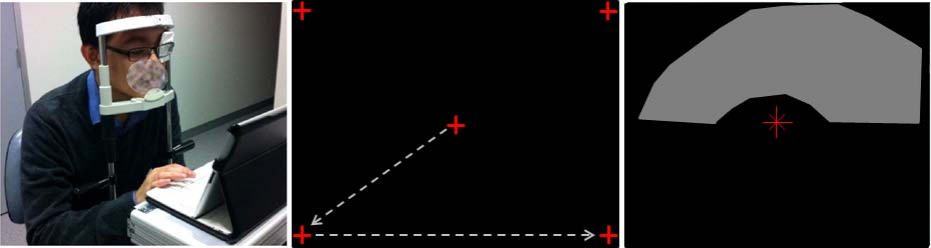
MRF performance was tested in five young participants who had normal vision. (**A**) The test site with chin and forehead rest and the observer fixating the iPad (at 33 cm) and responding on the keyboard. (**B**) The five fixation locations that are used sequentially during testing of the central 30° field. (**C**) A simulated visual field defect shown with a plastic overlay that reduced sensitivity in the superior arcuate region (*gray zone*).

As the screen of an iPad tablet measures 195 × 150 mm this restricts the tangent perimeter to some 15° × 12° eccentricity when viewed at 33 cm using central fixation. The peripheral visual field is tested by having the patient alter fixation to each corner of the tablet in turn (Fig. 4B). This is accompanied by a verbal instruction given by the iPad and by the fixation point moving to the desired corner with patients being instructed to track the fixation marker (note: no testing is undertaken during tracking). In this manner, peripheral locations can be tested out to 30° along the horizontal and 24° along the vertical. Although the verbal instruction appears straight forward a clinical trial needs to determine how naïve patients will cope with this instruction to change fixation and whether such testing can bias outcomes due to awareness of test spot location. A blind spot monitor is used when testing the central (15° × 12°) field. In the periphery the blind spot monitor cannot be applied due to its location and a voice instruction is given for the patient to fixate to the appropriate corner; this is replayed at regular intervals to facilitate compliance.

Stimulus size is varied with eccentricity to allow for the tangent effect of a planar tablet screen as well as to produce a fixed threshold across the central visual field.^17,18^ For this purpose and for the “free space” application, spot size was set approximately 15% larger than the scaling reported by Sloan^18^ that returns a fixed threshold and ranges from less than Goldmann size III at 3° eccentricity to just under Goldmann size V at 30° eccentricity. Testing beyond 17° uses a common size (17° size) adjusted for tangent effect. The luminance profile of all spots is ramped over 1 to 5 pixels to reduce edge detail.^17^ The spot configuration returns a single threshold value across the visual field due to constant spatial summation.^17^ This simplifies the test logic to a common PDF and optimizes the operational capacity of the iPad in the periphery, having the benefit that it increases the dynamic range of the iPad when testing scotoma located at noncentral points.

### Other Parameters

The MRF has an option for showing a red target. This has been configured to be isoluminant with the background within the constraints of the 8-bit control after measuring the spectral output of the red, green, and blue channels of the iPad as detailed by Metha et al.^10^ The saturation of the red channel is increased during this test with a concomitant reduction in green channel to retain isoluminance. We have expressed each level in terms of decibel equivalents on the 30-dB (white) scale to retain commonality between test modes.

A 300-ms stimulus duration is used followed by a random variable delay (700–1100 ms); any response made over this joint period is accepted. This is followed by a fixed prestimulus delay of 200 ms, where a response is not accepted. This timing is slightly longer than a conventional perimeter to allow for the hand movement needed in making a response. This timing means that screening of the entire visual field (66 points) should take approximately 100 seconds, whereas a full threshold would require approximately 4 to 5 minutes, depending on the nature of the field loss, time taken for refixation to corner locations and reliability of the patient (retest). A neighborhood logic is being developed to speed up testing further. Voice prompts for the test procedure have been programmed to be delivered in English by the tablet to guide the user throughout the test. The instructional language can be modified but this implementation is not yet available in the MRF. The user response to a stimulus is by screen touch (recommended touch zone is identified on the screen although touch anywhere is accepted) or it can be polled by a Bluetooth keyboard connected to the tablet. Here the patient is instructed to press the space bar on seeing a spot. We prefer the keyboard option to the screen response, as it provides better tactile feedback to the patient and keeps the screen from becoming stained by finger marks. Users can select which response option they would like to use before each test.

The MRF software was created in JavaScript and HTML5 on a Titanium Appcelerator platform (Mountain View, CA). This converts code into Apple Objective-C language that is compiled by an Xcode application (Apple, Inc.) for distribution as an app. Next we detail the proving trials of this development; the capacity of the MRF for diagnosis in a clinical population will be reported in another manuscript.

### Early Proving Trials

These trials were undertaken with approval from our institutional Human Research & Ethics Committee (HREC No. 15/1220H) and all volunteers provided informed consent prior to participating in accord with the tenets of the Declaration of Helsinki.

Five university students (22–28 years) volunteered as normal observers to evaluate the performance of the MRF. All had normal ocular and systemic health with visual acuity of 6/6 or better (with or without glasses). Participants had never performed perimetry before and were given two exposures to the MRF under close supervision to familiarize themselves with the testing procedure, the fixational changes required during the test and the voice instructions. These learning exposures were not scored and the results discarded.

Monocular testing was performed after a training period without supervision wearing the usual refractive correction and by occluding the fellow eye. Tests were on the eye with best acuity or a randomly selected eye when acuity was similar. A chin and head rest was used to limit head movements to the appropriate viewing distance (Fig. 4A). Care was taken to ensure that the iPad screen was clean and using a keyboard ensured that the screen was not tilted with respect to the viewing plane, as tilt has been shown to reduce target luminance.^1^

### Testing Procedures

All testing was performed in a dimly lit room (<4 lux from the iPad screen) except when the effect of ambient light was being evaluated. The screen brightness is set to maximum (100%) by the software and the iPad was turned on for at least 10 minutes prior to testing to ensure stability of luminous output.^1^ The repeatability of the MRF was considered by performing four visual field tests on each participant: these were repeated 1 week later. The results from the first (test 1, week 1) and the last tests (test 8, week 2) were used to determine the variability of MRF outcomes. Because ambient lighting during MRF testing may vary in remote locations, two levels of ambient light were considered: normal office light (600 lux in the horizontal plane of the iPad screen) and dim room light (<4 lux at the plane of the iPad). A test was also conducted on one observer with the reflection of a bright sunlit window covering half of the iPad screen to demonstrate the adverse effect that this can have on outcomes. The effect of pupillary myosis on thresholds was considered 30 minutes after of one drop of 1% pilocarpine (pupil < 2 mm: ave 1.1 [0.1] mm) in five participants. This was compared with the outcome returned from testing with a normal pupil (ave 6.4 mm).

Uncorrected refractive error can impact visual field results^19^ but the effect that it will have on the MRF is not clear as the spot size increases in peripheral locations, making it robust to refractive challenge. The fact that the MRF is administered at 33 cm means that a complete presbyope will experience 3 DS of blur without near correction. Many presbyopes will have multifocal lenses and at 33 cm the amount of blur may vary along different lines of sight through such lenses. So the effect of blur on MRF outcomes is an important consideration. Uncorrected refractive error was simulated 30 minutes after one drop of 0.5% tropicamide was instilled in the eye to neutralize accommodation and provide dilation. Trial lenses were used to simulate blur (plano and +3.00 DS) with the plano lens providing a +3 diopter (D) blur at the 33-cm viewing distance and the other providing working distance correction.

Scaling spot size to be 15% greater than the critical area for any eccentricity means we could expect little change in threshold with viewing distance variation, provided the spot remains larger than the critical area. This prospect was considered by testing at three viewing distances: 25, 33 (standard), and 75 cm. The effect of changing viewing distance from 33 to 25 cm is for a 1.7 times increase in spot area, whereas going from 33 to 75 cm gives a five times decrease in area. Our expectation is that the increase in area associated with the 25-cm viewing distance will have no effect on threshold as it is already larger than the critical area but the smaller spots associated with the 75-cm viewing distance should reduce threshold by approximately 6 dB.

Visual field defects were simulated by using multiple layers of an opaque plastic film that was cut in the form of a scotoma (central, arcuate, quadrant, hemifield). This was taped onto the iPad screen in the appropriate location prior to testing (Fig. 4C). The transparency was measured as producing a 7 to 9 dB reduction in light transmission. The simulated field losses were tested twice to determine point-wise variability in the region of the simulated scotoma.

### Data Analysis

Point-wise retinal thresholds were calculated by reflecting all left eye data in the vertical to produce equivalent right eye visual fields. Data were analyzed in terms of average thresholds across central (0°–6°) and peripheral (>6°) locations for the five subjects. Group data are shown as means [SEM] and were compared with a Student's *t*-test.

A Bland-Altman analysis was used to consider bias and 95% limits of agreement (LoA) when comparing retest data. Test variability was also considered in terms of the coefficient of repeatability (CR = SD/geometric mean).^20^ For simulated defects, abnormal locations were identified as being beyond the 97.5% confidence limit (CL) of normal values. A separate Bland-Altman analysis was performed for these abnormal thresholds to determine their CR.

## Results

**Figure 5:**
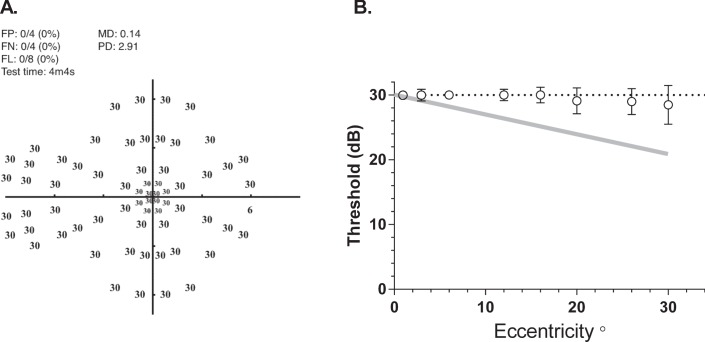
Thresholds measured with the MRF. (**A**) Representative thresholds (dB) for one young (22-year old) participant who needed just over 4 minutes to complete. (**B**) Average [SEM] group threshold (*circles*) for five participants as a function of eccentricity. Note how increasing the size of the target with eccentricity has flattened the usual ‘Hill of Vision' that is found when testing with a size III target (*gray line*: −3 dB per 10°).

Figure 5A shows MRF thresholds returned by one observer where it is evident that spot-size scaling returns a constant threshold of 30 dB across the visual field. This is confirmed by the group average data (29.6 dB [0.12]) shown in Figure 5B. MRF thresholds do not give the eccentricity related drop off found when perimetry is performed using a Goldmann size III stimulus (gray line, Fig. 5B). Average test duration for the MRF in our young participants was 4.1 [0.2] minutes across all tests in the normal state. This test time includes the time required for the iPad to play voice prompts and for the person to move their fixation when testing in the periphery (Fig. 4B).

**Figure 6:**
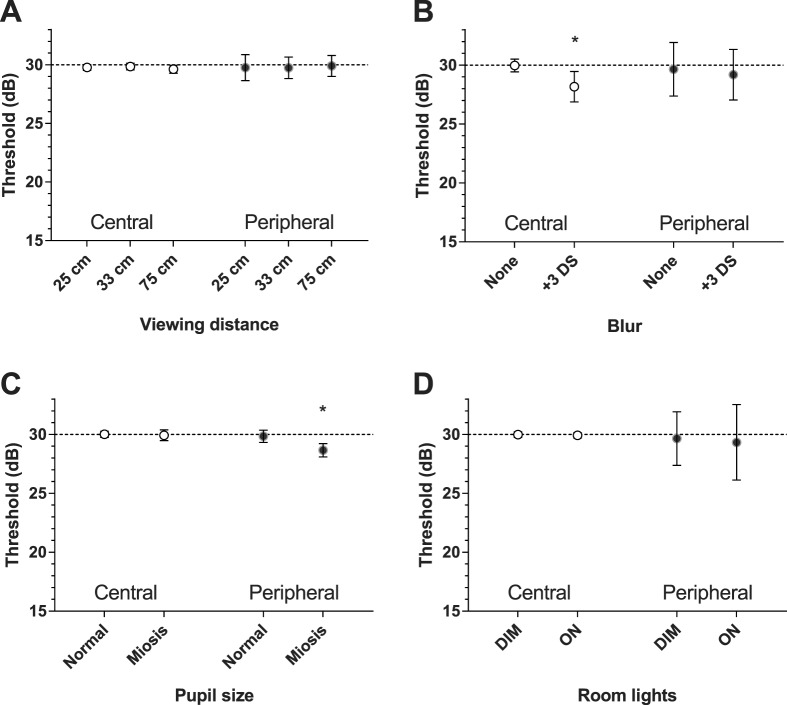
The effect that varying ambient test conditions has on average threshold (SEM) across central (0°–6°) and peripheral (>6°) locations of our participants. (A) Variation in viewing distance (25, 33, and 75 cm) has little impact on the average threshold. (B) Blur produces a significant reduction in average threshold (−1.5 dB) at central locations only. (C) Miotic pupils result in a significant reduction in average threshold (−1.2 dB) at peripheral locations. (D) Variation in ambient room lighting (600 and 4 Lux) has little impact on central or peripheral threshold. Where *error bars* are not visible, they are smaller than the *symbol. Asterisk* identifies significant change (*P* < 0.05).

Figure 6 summarizes the average outcomes for central (0°–6°) and peripheral (>6°) locations separately in the presence of blur, myosis, variable viewing distance, and change in ambient light. Blur produced a significant reduction (−1.7 dB) in central threshold only (29.9 [0.27] vs. 28.2 [0.64], *P* = 0.01), whereas myosis gave a significant reduction (−1.2 dB) at peripheral locations (29.8 [0.26] vs. 28.6 [0.28], *P* = 0.04). Changes in viewing distance and variations in ambient light did not significantly alter thresholds at any location (Fig. 5).

**Figure 7:**
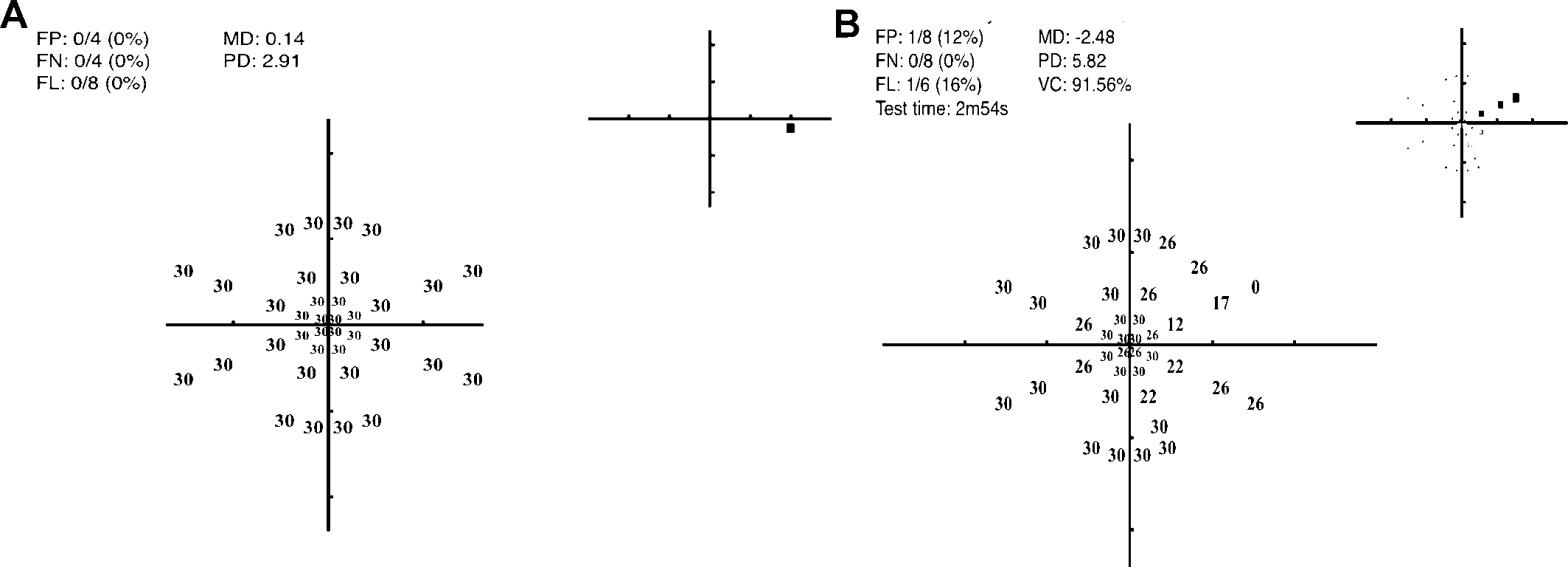
The adverse effect that a bright screen reflection can have on thresholds. The panel on the *left* was obtained when the observer was tested in a darkened room: it shows the decibel values with a normal MD (0.14) and PD (2.9). The panel on the *right* shows the result for the same observer (dB values and *gray scale inset*) when tested with the reflection of a bright window visible in the *right upper half* of the screen. Here the mean defect (MD) was −2.48 and pattern defect (PD) 5.82. Note, the test pattern is a subset of the full 66-point grid that takes approximately 3 minutes for completion.

Figure 7 shows the threshold data of one observer obtained in a dimly lit room without reflection (left panel) and when testing was undertaken with a bright window reflected in the right hand side of the screen (right panel). The presence of this veiling glare reduced MD by 2.6 dB.

**Figure 8:**
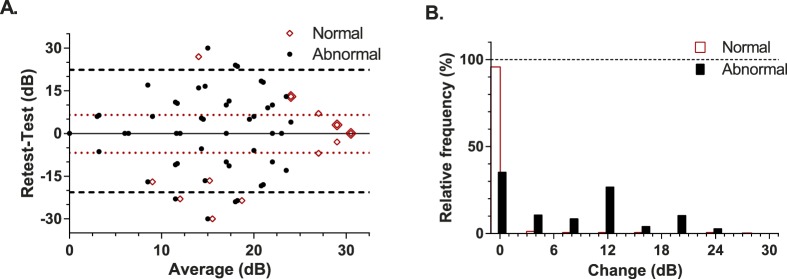
Test-retest performance in our participants. (A) Bland-Altman analysis with the *red unfilled diamonds* showing normal variability and the *horizontal dotted lines* giving the 95% LoA for these data. As many points are superimposed on a common location these locations have been indicated by the *large diamond* overlay. The *filled circles* show the retest variability at abnormal locations (<25 dB) in the presence of a simulated scotoma. The *horizontal dashed lines* mark the 95% LoA for these abnormal data. Data have been jittered along the *x* axis for clarity. (B) The frequency of different amounts of change encountered at retest plotted for normal (*unfilled bars*) and regions of simulated scotoma (*filled bars*). Frequency has been expressed as a percentage of the total test point population.

**Figure 9:**
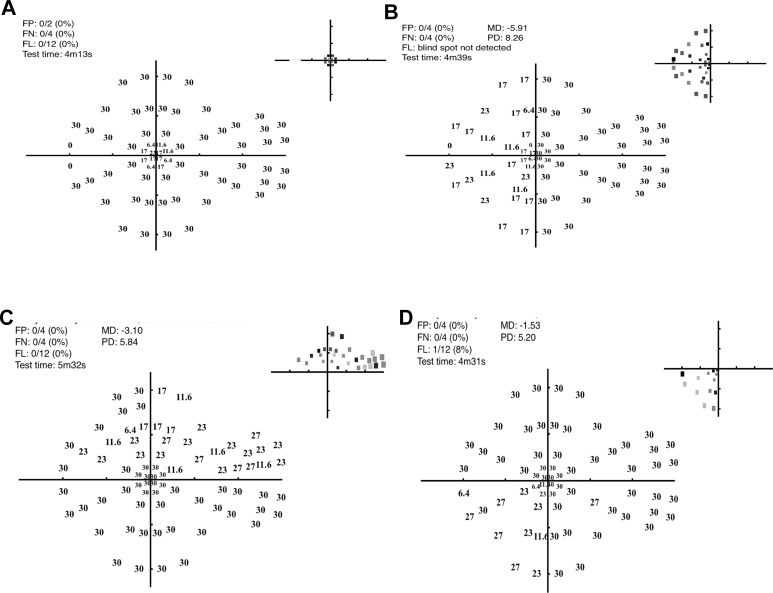
Representative thresholds (decibels and *gray scale inset* at *top right* of each panel) for one participant in the presence of four different simulated scotoma (as per Fig. 4C). (A) Central scotoma, (B) hemianopia, (C) superior arcuate defect, and (D) quadrantic defect. The *gray scale* plots in the *top right corner* of each plot code the abnormal locations based upon the normal probability of Figure 8B.

The retest performance of the MRF is shown in the upper panel of Figure 8 as a Bland-Altman plot: here a symbol can represent multiple data. It is apparent that both threshold and repeatability varies depending on the nature of the location (normal versus abnormal). Normal locations (unfilled diamonds) tend to give stable outcomes that are greater than 25 dB (97.5% CL) and have tight limits of agreement (95%: −6.8 to 6.5 dB; dotted horizontal). The coefficient of repeatability for normal locations of the MRF was 9.6% (SD ∼ 2.9 dB). Abnormal points lying within simulated scotoma (filled circles Fig. 8) generate many locations with low sensitivity (average 16.6 dB) and give larger limits of agreement (95%: −21 to 22 dB; dashed horizontal) than do normal data. The coefficient of repeatability for abnormal locations in regions of simulated loss was 48.1% (SD ∼8.0 dB). Figure 8B is a frequency histogram that shows the amount of change found at retest for normal or abnormal locations (<25 dB, see earlier) relative to the total number of points. Ninety-seven percent of normal locations vary by less than 5 dB at retest compared with 48% of abnormal locations.

**Figure 10:**
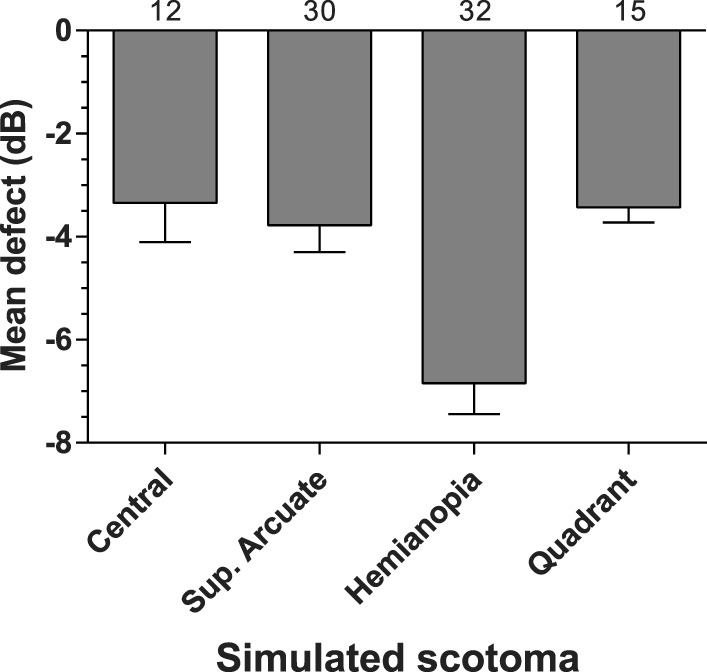
Group average MD (SEM) for four types of simulated scotoma labelled on the *x* axis. The same group returned a MD of 0.26 under normal test conditions. The numbers at the *top* of each *bar* show the median number of abnormal test locations in the simulation.

Figures 9 and 10 consider the capacity of the MRF to detect simulated scotoma. Figures 9A through 9D show representative data for four different scotoma simulations. Note that the testing takes up to 1.4 minutes longer (4.2–5.5 minutes) in the presence of a scotoma compared with testing of normal locations (4.1 minutes) due to the checking procedure that guards against lapses of concentration (see earlier). Figure 10 shows the average mean defect (MD)^9^ for the simulated conditions across all participants, this ranges from −3.3 to −6.8 dB. The numeric at the top of each bar of Figure 10 gives the median number of abnormal (<25 dB) data points found with each simulation.

## Discussion

We calibrated the luminous output of two tablet devices and find that although they return different absolute luminances their operating ranges are similar at approximately 31 dB and should be adequate to undertake human threshold estimates across the visual field. This supports the findings of Wu et al.^4^ who compared a tablet against a commercial microperimeter using a background luminance of 1.27 cd.m^−2^. That group found that the tablet gave reliable threshold estimates of comparable magnitude, which is not surprising given that the stimulus locations were aligned between the two instruments, as was the background and maximum spot luminance, so concordance would have been optimized with differences arising from patient and test logic variability. In our implementation, the test logic differs and MRF locations vary from the HFA test grid, however, we have chosen a background luminance (5 cd.m^−2^) that should compare with the HFA 35-dB level in terms of human contrast thresholds. We believe that doing so should yield a strong correlation between the HFA and MRF, which is the topic of a fellow paper.

The MRF has been designed to be fast, by adopting a three step decision tree generated by Bayes forecasting principles.^12–14^ Sita (fast and standard) make use of such principles post hoc to a staircase to return the most likely threshold, whereas we apply them during the test as enunciated by King-Smith et al.^14^ to efficiently guide the threshold to the next most likely value. This yields a very rapid test and we find that participants with normal vision complete testing of 66 locations in 4.1 minutes, which includes time for fixation changes. Our approach to thresholding is unusual in that we presume that the patient is reliable. We show from our data that 97% of all test points in normal locations yield little change (0–5 dB) from initial tested values supporting this presumption. In order to allow for lapses of concentration in these otherwise reliable patients, we implement a neighborhood logic that triggers retesting at a location whenever it is removed from the value expected from its neighbors. That 97% of locations are stable confirms that this logic works well, although it does require additional time for retesting (∼6 seconds per location). Such neighborhood checking, will increase test times in scotomatous regions especially at the edge of a scotoma where sensitivity changes are found. Not surprisingly, we find an increase in test time in patients having simulated scotomas by approximately 1 minute. This strategy needs a clinical trial to evaluate its usefulness but from our simulation data, its performance looks promising.

The MRF has adopted locations that can detect common causes of vision loss (diabetes, AMD, neurological, glaucoma) and our simulation shows that it does so even with mild defects. This should yield a good test for general clinical populations. It is our belief that the test grid and test logic can be further optimized for specific patient profiles (e.g., retinal disease) or for monitoring progression in subpopulations of clinical patients, an issue that is in need of further research and development.

We find that the MRF outcomes are surprisingly robust to environmental factors (myosis, blur, viewing distance, and ambient illumination). Blur and myosis were found to have minor (∼1.5 dB) effect on threshold whereas viewing distance had none that we could expose. We feel that this robustness has been created by the variable spot size which is 15% larger than the critical area found by Sloan.^15^ This means that the test returns a constant threshold at all eccentricities and the larger sizes will make the test robust to blur and changes in pupillary diameter. It will also make the test robust to variable viewing distance especially at near but we do not understand why threshold did not change at the 75-cm viewing distance, as we expected a 6-dB decrease (see earlier). Perhaps the more central location of spots produced by the greater viewing distance has offset any decrease from the smaller size, this possibility needs clarification.

The design features of the MRF were developed to facilitate home testing and it appears that they have achieved this goal. That ambient illumination had no effect on threshold indicates that performance is on the Weber slope and returns a constant threshold in the presence of greater ambient light. Nevertheless, in order to return the largest dynamic range and to prevent unwanted reflections that reduce spot visibility, we recommend that the test be performed in a dim and evenly lit room with no direct reflections of doorways or windows on the screen. That changes to viewing distance have no impact on threshold is also a useful design feature for the free space home-monitoring environment. The spot size was intentionally made 15% larger than the critical area to allow for variation in viewing distance. This larger size will remain within the critical area for up to an 11-cm increase in viewing distance (from 33 to 44 cm), which we feel provides adequate reserve for home monitoring purposes. Nevertheless, we recommend that a simple solution, like a string, be adopted to set the proper viewing distance (33 cm) for testing. The impact that free space viewing can have on thresholds awaits a clinical trial for quantification.

The extension of a portable tablet device to peripheral visual field testing, as detailed here, has the potential to allow detection and management of eye and brain disease in communities where access to traditional field testing machines is limited. Furthermore, it will allow future investigation into the use of such testing devices in terms of home monitoring. Home visual field monitoring can complement existing technologies such as HFA and extend clinical visual field testing by reducing resource burden on clinics and by allowing frequent field testing of patients to yield earlier detection of visual field change.^21^ We are presently modelling the benefits that can be expected from home testing on early detection of change in order to develop an efficient approach that can be implemented for this purpose.

This manuscript has detailed the development of the MRF and has reported a simulation that suggests that the device has sound potential to find early defects with small MD values (−3.3 dB; Fig. 10). To fully understand the implementation of this device in clinical settings, further investigation is required. In the present implementation we attempt to reduce fixation loss in peripheral regions with regular voice prompts played by the device that reminds participants to maintain fixation. However, this is not a robust solution. Despite this limitation, in the current sample of participants under conditions of simulated scotoma, the MRF was able to detect retinal sensitivity loss and to do so reliably and repeatedly as indicated by the test-retest analysis. Its concordance with a HFA in a clinical population will be the subject of a future manuscript.
